# Supplementary material for: Genome-Wide Analysis of Sugar Transporters Identifies the gtsA Gene for Glucose Transportation in Pseudomonas stutzeri A1501
Source: Microorganisms. 2020 Apr 19;8(4):592. doi: 10.3390/microorganisms8040592 (PMC7232493; doi:10.3390/microorganisms8040592)
Supplement: Supplementary file 1 [file microorganisms-08-00592-s001.pdf]

Table 1. Primers for RT-PCR and qPCR.

Primer name	Sequence (5′-3′)	Amplified fragmenta
testF1	TTCGTCTCGCTCAACGACAA	1605 bp *gtsA-up* and *Cat* internal fragment
testR1	TCACCAGCTCACCGTCTTTC	
testF2	TTCTTGCCCGCCTGATGAAT	1621 bp *Cat* and *gtsA-down* internal fragment
testR2	CAGCAACATCACGATCAGCG	
testF3	TTGTAAAACGACGGCCAGTG	1498 bp internal fragment with *gtsA*
testR3	CTCACTCATTAGGCACCCCA	
gtsA-F	GAAGACCCTCGACGAGTTCT	1141 bp *gtsA* and *gtsB* internal fragment
gtsB-R	AGGTAGATGGTGCGGATCAG	
gtsB-F	CTGCTGTCGTTCACCAACTC	1017 bp *gtsB* and *gtsC* internal fragment
gtsC-R	ACGATCTTCACCGAGTTCCA	
PST0988-F	TGAAACTGAATGAAGCCGCC	202 bp internal fragment
PST0988-R	ATTTCGGCGAACACCTTGTC	
PST1574-F	CCTGGGCATCATGTCATTGG	239 bp internal fragment
PST1574-R	CTACACCGATCAACAGCGTG	
PST1604-F	AGCATCATCTGGGGTATCGG	195 bp internal fragment
PST1604-R	GCTGTACAACCCGTAGACCA	
PST1613-F	CTATCCACGGGGCTTTCTGA	160 bp internal fragment
PST1613-R	CAGTACCTTCGATTGCACGG	
PST1972-F	TGTGCTGGTACCGAACTTCT	190 bp internal fragment
PST1972-R	CATCGCCAGGCTCAAAAGG	
PST2191-F	GATCTTCTGGCACCTGACCT	155 bp internal fragment
PST2191-R	TGTAGATCAGGTACGCGAGG	
PST2437-F	ATCCACCCTGATGAACTGCA	218 bp internal fragment
PST2437-R	CCACCTCCTCCTCGATCTTC	
PST2438-F	GCGTTCACCACACTGTTCTT	249 bp internal fragment
PST2438-R	ATTGACCAGGTTGTTCAGCG	
PST2439-F	CAGTGCGTTGATGATCCTCG	217 bp internal fragment
PST2439-R	CAGCTCGGAGTACAGGTAGG	
PST2440-F	ACCCACGACTTGCAGGATAA	182 bp internal fragment

PST2440-R	GCTCGACCATCTTCTGCAAG	
PST2907-F	CAGAAATTCATCCTCGGGCG	155 bp internal fragment
PST2907-R	TCGGAGATCACCAGAATCGC	
PST3484-F	TCTTCCAAATGTTTCCGCCG	161 bp internal fragment
PST3484-R	GTAGCCCTTGATGGTCCAGA	
PST3581-F	GATTCTCAAGGTGCTGCTGG	161 bp internal fragment
PST3581-R	TCACGTCGCTCATTTTGTGG	
16S-F	CCTACGGGAGGCAGCAG	150 bp internal fragment
16S-R	ATTACCGCGGCTGCTGG	

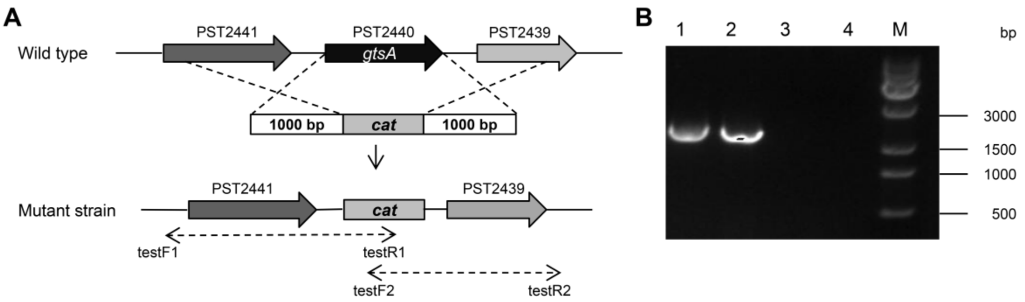

Figure S1. Construction and validation of *gtsA* deletion mutant. (A) Schematic representation of the *gtsA* deletion mutant generated by replacing the *gtsA* region with the chloramphenicol resistance gene *cat* (Cm^r^). The primer pairs testF1–testR1 and testF2–testR2 were used to analyze the *gtsA* deletion as indicated by arrows and the corresponding sequences are shown in Table S1. (B) Validation of *gtsA* deletion mutant by colony PCR. The testF1–testR1 (lanes 2 and 4) and testF2–testR2 (lanes 1 and 3) junctions were amplified using wild type strain (lanes 3 and 4) and *gtsA* deletion mutant (lanes 1 and 2) as the template. Lane M, 15 kb plus DNA ladder, and the sizes of the molecular markers are indicated at the side in bp.

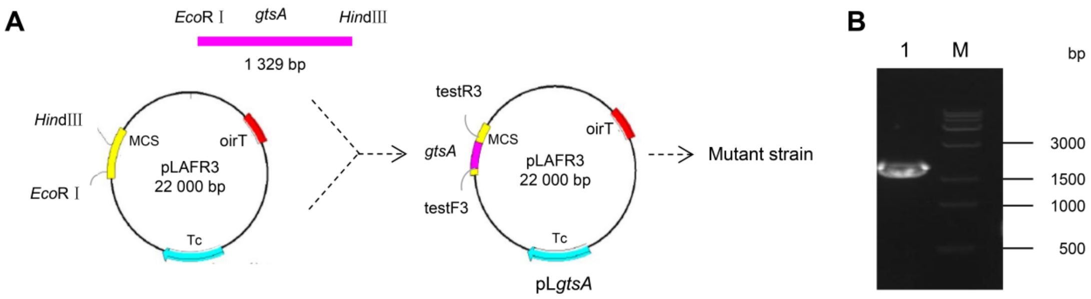

Figure S2. Construction and validation of Δ*gtsA* (pL*gtsA*). (A) Schematic representation of the Δ*gtsA* (pL*gtsA*) generated by introducing pL*gtsA* into the *gtsA* deletion mutant. The primer pair testF3–testR3 was used to analyze the Δ*gtsA* (pL*gtsA*) and the corresponding sequences are shown in Table S1. (B) Validation of Δ*gtsA* (pL*gtsA*) by colony PCR (lane 1). Lane M, 15 kb plus DNA ladder, and the sizes of the molecular markers are indicated at the side in bp.

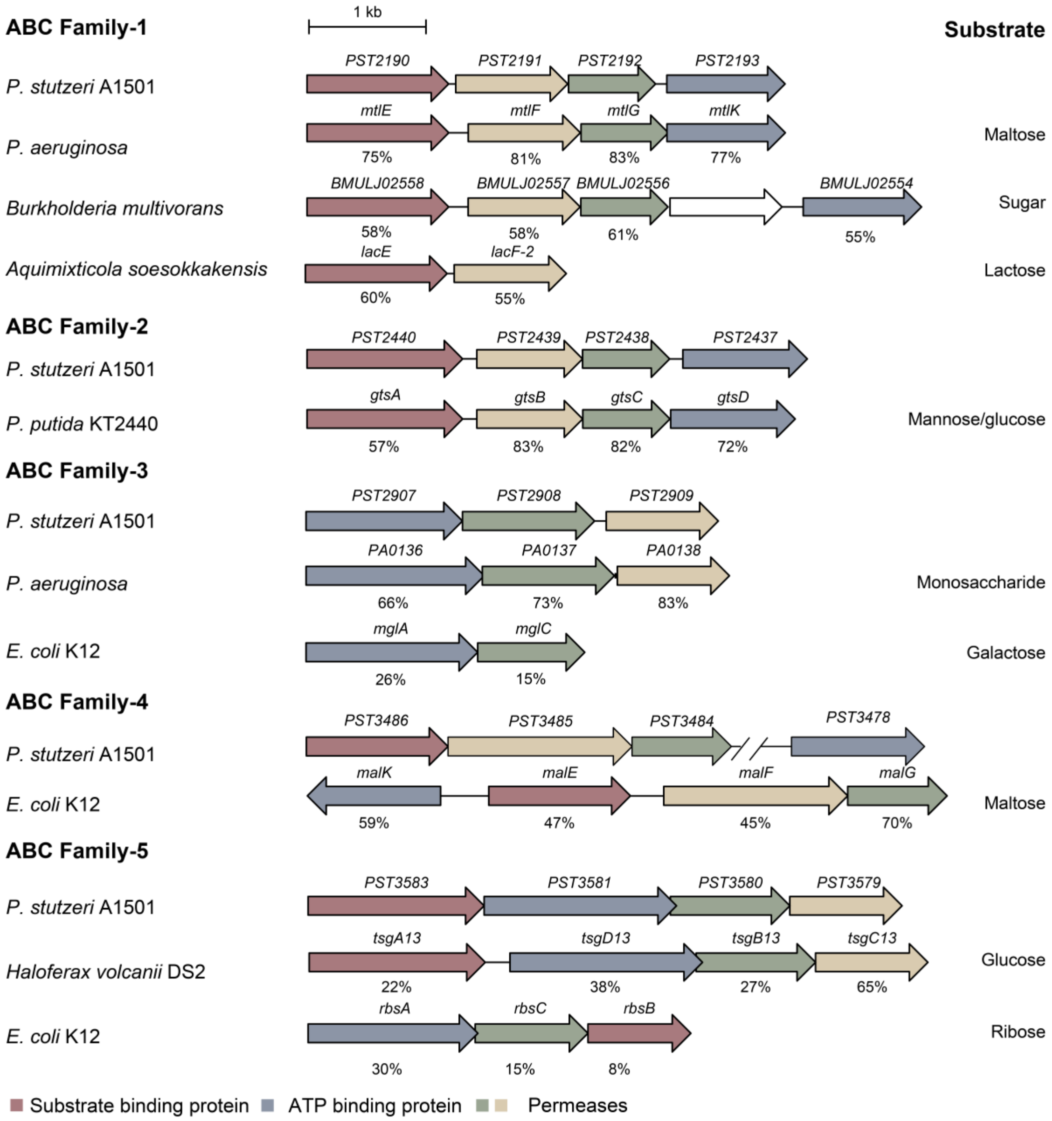
ABC Family-1
1 kb
Substrate
P. stutzeri A1501
PST2190
PST2191
PST2192
PST2193
P. aeruginosa
mtlE
mtlF
mtlG
mtlK
75%
81%
83%
77%
Maltose
Burkholderia multivorans
BMULJ02558
BMULJ02557
BMULJ02556
BMULJ02554
58%
58%
61%
55%
Sugar
Aquimixticola soesokkakensis
lacE
lacF-2
60%
55%
Lactose
ABC Family-2
P. stutzeri A1501
PST2440
PST2439
PST2438
PST2437
P. putida KT2440
gtsA
gtsB
gtsC
gtsD
57%
83%
82%
72%
Mannose/glucose
ABC Family-3
P. stutzeri A1501
PST2907
PST2908
PST2909
P. aeruginosa
PA0136
PA0137
PA0138
66%
73%
83%
Monosaccharide
E. coli K12
mglA
mglC
26%
15%
Galactose
ABC Family-4
P. stutzeri A1501
PST3486
PST3485
PST3484
PST3478
E. coli K12
malK
malE
malF
malG
59%
47%
45%
70%
Maltose
ABC Family-5
P. stutzeri A1501
PST3583
PST3581
PST3580
PST3579
Haloferax volcanii DS2
tsgA13
tsgD13
tsgB13
tsgC13
22%
38%
27%
65%
Glucose
E. coli K12
rbsA
rbsC
rbsB
30%
15%
8%
Ribose
Substrate binding protein
ATP binding protein
Permeases

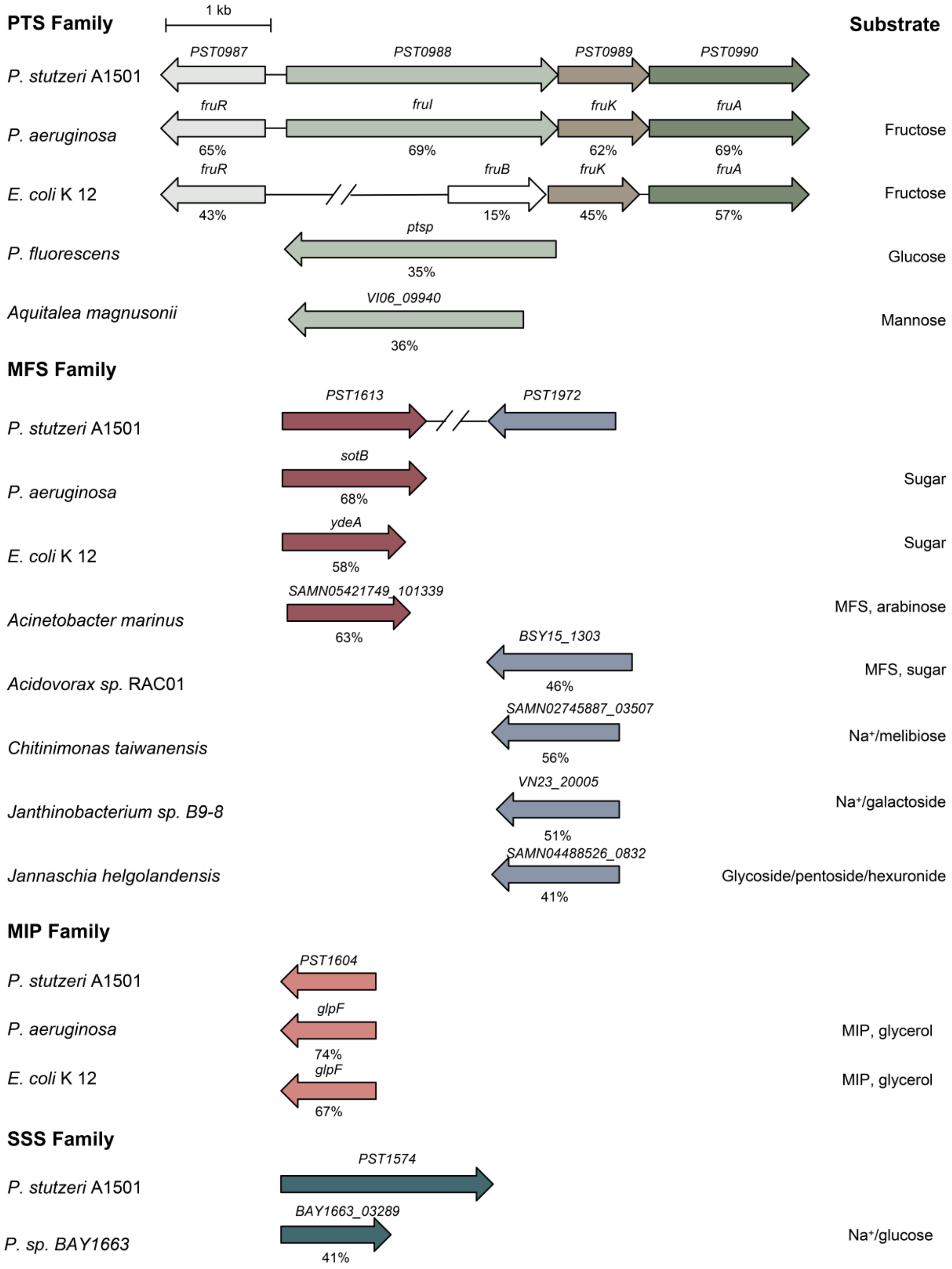

Figure S3. Protein sequence alignment of sugar transport system components of *P. stutzeri* A1501 and other microorganisms. The arrows indicate annotated genes and predicted open reading frames length and transcription orientation.

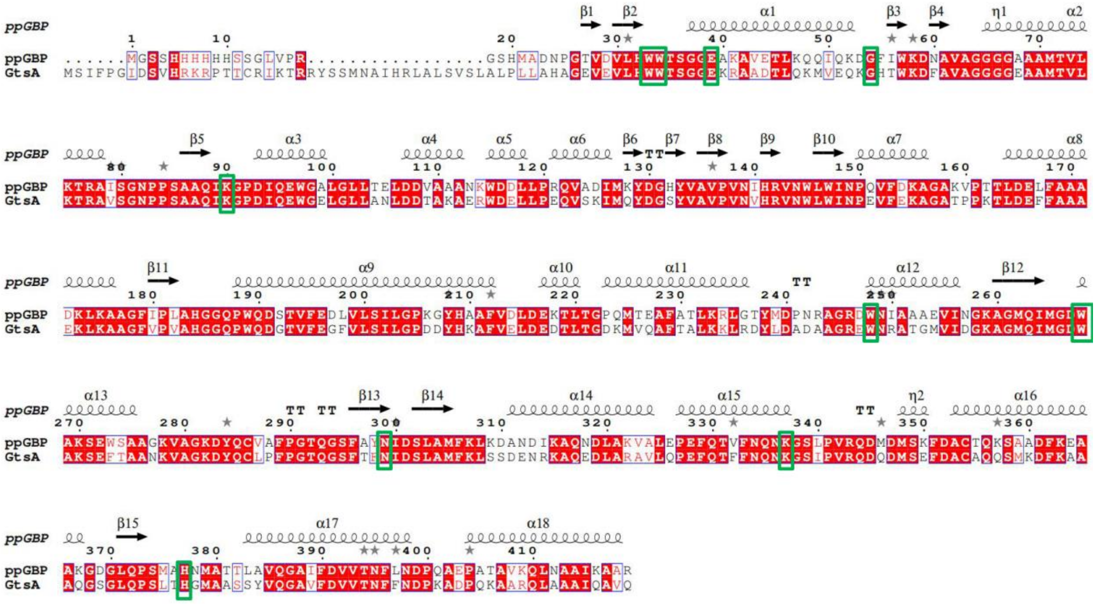

Figure S4. Amino acid sequence alignment of GtsA with ppGBP. The alignment was conducted using ClustalW and is presented along with secondary structural elements of ppGBP on the top using ESPript 3.0 (http://espript.ibcp.fr/ESPript/ESPript/). GtsA contains all glucose-binding residues in ppGBP (green box).
